# Complete transposition of the great arteries with borderline tricuspid valve: how small is too small? A case report

**DOI:** 10.1093/ehjcr/ytag432

**Published:** 2026-06-09

**Authors:** Cong Chao, Pengchao Xing, Silin Pan, Rui Chen

**Affiliations:** Qingdao Women and Children’s Hospital, Qingdao University, No.6 Tongfu Road, Qingdao City, Shandong Province 266000, P.R. China; Qingdao Women and Children’s Hospital, Qingdao University, No.6 Tongfu Road, Qingdao City, Shandong Province 266000, P.R. China; Qingdao Women and Children’s Hospital, Qingdao University, No.6 Tongfu Road, Qingdao City, Shandong Province 266000, P.R. China; Qingdao Women and Children’s Hospital, Qingdao University, No.6 Tongfu Road, Qingdao City, Shandong Province 266000, P.R. China

**Keywords:** Complete transposition of the great arteries, Ventricular septal defect, Arterial switch operation, Right ventricular dysfunction, Z-score, Case report

## Abstract

**Background:**

Previous studies have mostly focused on the maintenance of left ventricular function after corrective surgery for transposition of the great arteries (TGA), with limited research on the recovery of postoperative right ventricular dysfunction.

**Case summary:**

In April 2025, our hospital admitted a neonate with complete transposition of the great arteries complicated with ventricular septal defect (TGA/VSD) and hypoplastic right ventricle (RV). The arterial switch operation (ASO) was performed on the eighth day after birth, followed by delayed sternal closure. The neonate developed refractory right ventricular dysfunction and was treated with inotropic agents, diuretics, anti-inflammatory agents, peritoneal dialysis support, inhaled nitric oxide, and thoracic drainage. The patient recovered successfully, and follow-up echocardiography at 6 months postoperatively showed normalization of right ventricular function.

**Discussion:**

We conclude that for patients with TGA/VSD, preoperative evaluation should not only focus on left ventricular development but also fully emphasize the status of right ventricular development. A borderline RV is not an absolute contraindication to ASO, although it may complicate postoperative management and necessitates long-term close follow-up of right ventricular function.

Learning points

- A borderline-but-viable right ventricle (RV) can be distinguished from true hypoplasia by intact tricuspid valve, normal leaflet excursion, no RV-dependent coronary circulation, tricuspid Z-score >−3, tricuspid-to-mitral ratio >0.5, and no RV hypertrophy.
- A borderline RV is not an absolute contraindication to arterial switch operation when comprehensive echocardiographic criteria are satisfied.

## Introduction

Transposition of the great arteries (TGA) is a common cyanotic congenital heart disease with a global incidence of ∼2.95 per 10 000 live births.^1^ Without surgical intervention, 50% of TGA infants die in the neonatal period, and the 1 year mortality rate is as high as 90%.^2^ Arterial switch operation (ASO) can anatomically correct the malformation and is the preferred surgical method for TGA.^3,4^ Low cardiac output syndrome is a common complication after ASO. Previous studies and clinical practices have excessively focused on left ventricular function maintenance in TGA infants after ASO while failing to preserve right ventricular function. Existing studies have shown that right ventricular dysfunction has predictive value for increased risk of cardiovascular events.^5^ Our hospital admitted a neonate with TGA/ventricular septal defect (VSD) in April 2025, who had a borderline right ventricle (RV) before surgery and developed refractory right ventricular dysfunction after surgery. This case is now reported as follows.

## Summary figure

**Figure:**
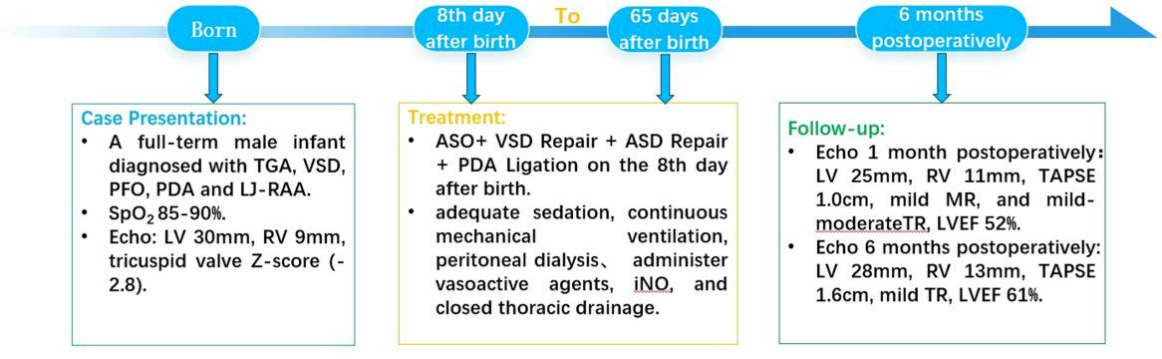


## Case presentation

**Figure 1:**
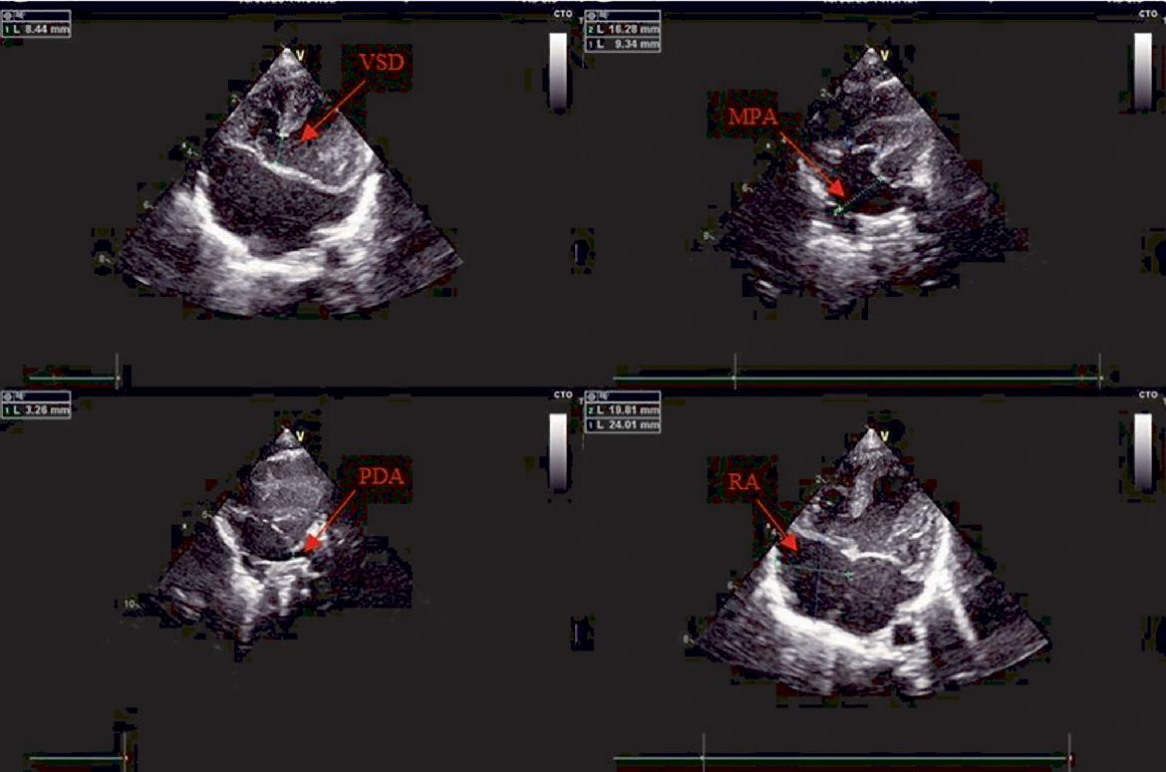
Preoperative echocardiogram. Note: VSD = ventricular septal defect. PDA = patent ductus arteriosus; MPA = main pulmonary artery; RA = right atrium.

A male neonate, 16 min old, was admitted to the hospital due to prenatal ultrasound findings of complete transposition of the great arteries for >3 months. The neonate was the first child of a gravida 3, para 1 mother, born via caesarean section at 39^+1^ weeks of gestation (birth weight 4020 g, Apgar scores 9 at 1, 5, and 10 min). After birth, he was transferred to the cardiac intensive care unit (CICU) through the prenatal–postnatal integrated care pathway. Physical examination: Peripheral capillary oxygen saturation (SpO_2_) was 85%–90% without oxygen inhalation, accompanied by tachypnoea. No obvious heart murmur was heard, and capillary refill time (CRT) was <3 s. Auxiliary examinations: Echocardiography (*Figure 1*) showed the following: (i) Atrial situs solitus, bilateral atrial appendages seemed to be located on the same side of the arteries, ventricular D-loop, atrioventricular concordance. (ii) The left atrium (LA) and left ventricle (LV) were enlarged, RV was small, the tricuspid valve Z-score was −2.8, and the right atrium (RA) size was basically normal. (iii) The aorta originated from the anatomical RV and was located anteriorly, with both the annulus and ascending aorta diameters of ∼8 mm. The main pulmonary artery originated from the anatomical LV and was located posteriorly, with a pulmonary valve annulus of 9 mm, tricuspid pulmonary valve, no fibrous connection between the pulmonary valve and mitral valve, and visible muscular conus. The two great arteries were arranged in parallel. (iv) Patent ductus arteriosus (PDA) was 3.3 mm with continuous bidirectional shunt. (v) Patent foramen ovale (PFO) was 2 mm with left-to-right shunt. A perimembranous inflow VSD (size ∼8.4 mm) was found with bidirectional low-velocity shunt.

**Figure 2:**
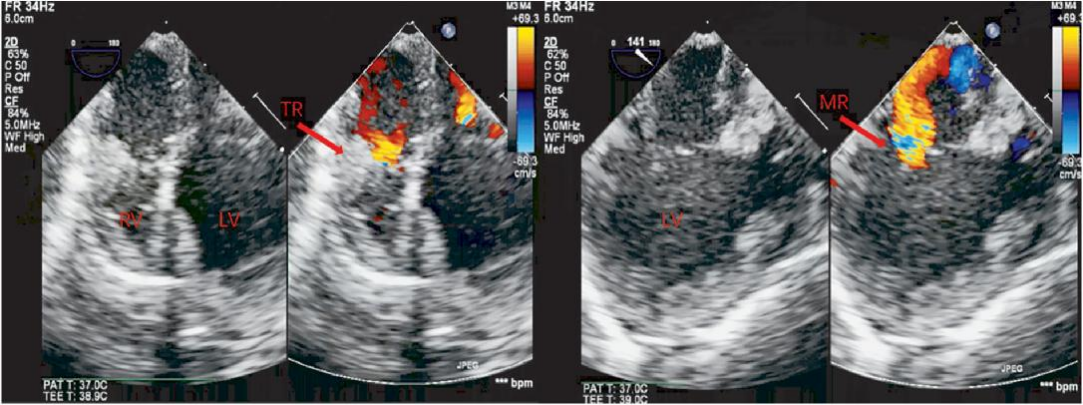
Intraoperative transoesophageal echocardiography. Note: LV = left ventricle; RV = right ventricle; TR = tricuspid regurgitation; MR = mitral regurgitation.

On Postnatal Day 8, he underwent ASO + VSD repair + atrial septal defect (ASD) repair + PDA division under general anaesthesia and cardiopulmonary bypass (CPB). Aortic cross-clamp time is 183 min, and CPB time is 303 min. Intraoperatively, marked left ventricular enlargement, normal tricuspid valve structure, small tricuspid orifice, and RV were noted. Post-ASO, obvious myocardial oedema and depressed cardiac function were observed; intraoperative exploration confirmed satisfactory filling of the left and right coronary arteries and their branches. Transoesophageal echocardiography (TEE) showed marked left ventricular enlargement, small RV, and moderate–severe tricuspid regurgitation (*Figure 2*). Haemodynamics is SpO_2_ 80%–90% (fraction of inspired oxygen FiO_2_ 100%), central venous pressure (CVP) is 15–20 cmH_2_O, and blood pressure (BP) is 40–55/25–35 mmHg. After 70 min of CPB support, cardiac function improved, and CPB was weaned. Postoperatively, SpO_2_ is 90% (FiO_2_ 80%), CVP is 13 cmH_2_O, and BP is 60/35 mmHg. He was transferred to CICU with delayed sternal closure.

**Figure 3:**
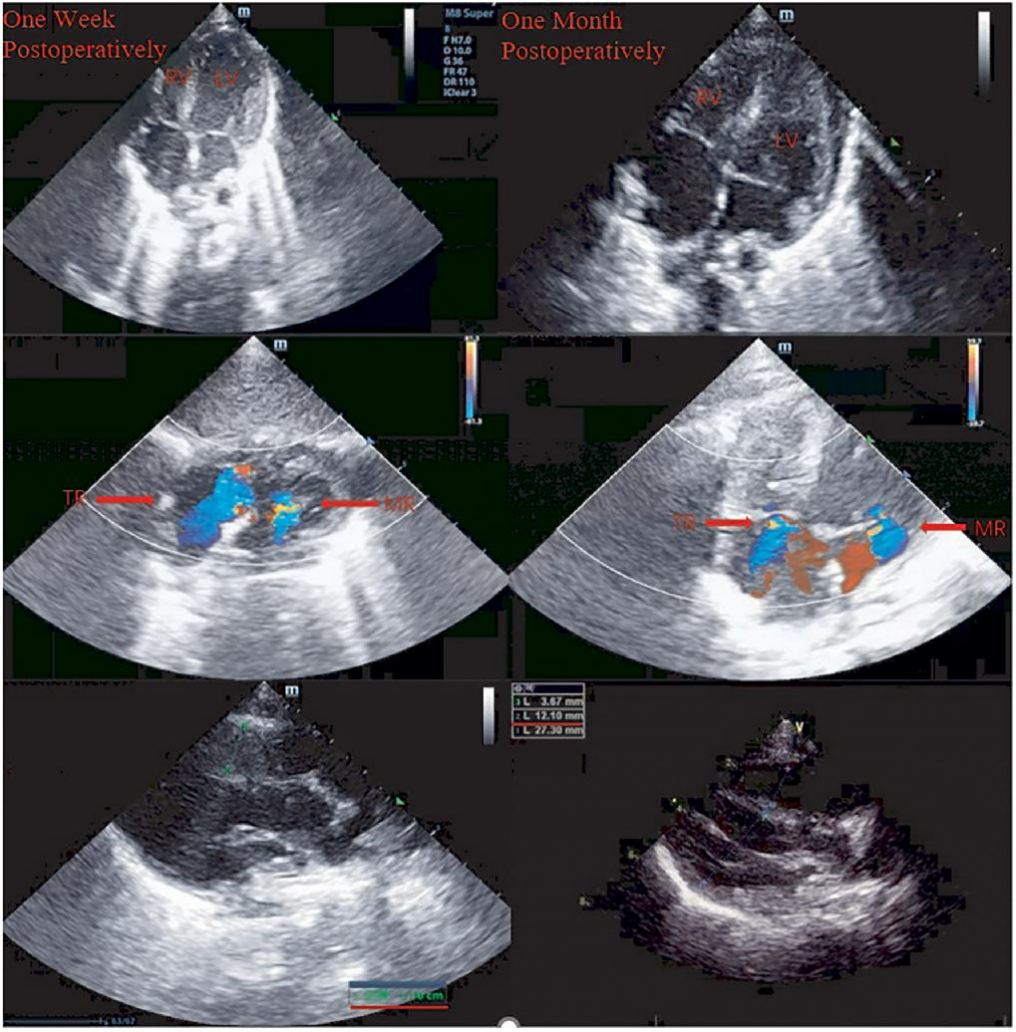
Comparison of echocardiography between 1 week and 1 month postoperatively. Note: LV = left ventricle; RV = right ventricle; TR = tricuspid regurgitation; MR = mitral regurgitation.

Postoperatively, he received sedation, inotropes (epinephrine, dobutamine, dopamine, milrinone), vasopressors (norepinephrine, vasopressin), diuretics (torsemide), hydrocortisone, and 20 ppm nitric oxide (NO) for oxygenation. Severe myocardial oedema delayed sternal closure until Postoperative Day 10. Persistent oliguria resolved after 25 days of peritoneal dialysis. Low oxygenation was managed with NO for 14 days followed by oral sildenafil for 2 months. Supraventricular tachycardia on Day 5 resolved with amiodarone infusion for 2 days. Bilateral chylothorax on Day 7 was treated with chest tubes, which were removed on Day 30; he was fed medium-chain triglyceride formula until discharge. Mechanical ventilation was maintained for 33 days, and the CICU stay lasted 55 days. Regular postoperative echocardiography showed gradual improvement in right ventricular function/size, with TAPSE normalized at 6 month follow-up (*Table 1* and *Figure 3*).

**Table 1:** Preoperative and postoperative follow-up echocardiographic parameters of the patient

	Preoperative	Postoperative Day 0	Postoperative Month 1	Postoperative Month 3	Postoperative Month 6
LVIDd	30 mm	35 mm	25 mm	27 mm	28 mm
AO	8 mm	11 mm	10 mm	12 mm	16 mm
MPA	16 mm	7 mm	7 mm	9 mm	11 mm
RA	24*20 mm	16*12 mm	18*16 mm	23*19 mm	26*22 mm
RV	9 mm	6 mm	11 mm	12 mm	13 mm
RVAW	3 mm	3 mm	3 mm	2.5 mm	3 mm
LVAW	3 mm	3 mm	3 mm	3.5 mm	3.2 mm
IVS	3 mm	3 mm	3 mm	3.5 mm	3.2 mm
LVEDV	35 mL	—	20 mL	27 mL	31 mL
TAPSE	15 mm	5 mm	10 mm	13 mm	16 mm
LVEF	64%	35%	50%	64%	61%

Note: LVIDd, left ventricular internal diameter in diastole; AO, aorta; MPA, main pulmonary artery; RA, right atrium; RV, right ventricle; RVAW, right ventricular anterior wall; LVAW, left ventricular anterior wall; IVS, interventricular septum; LVEDV, left ventricular end-diastolic volume; TAPSE, tricuspid annular plane systolic excursion; LVEF, left ventricular ejection fraction.

## Discussion

TGA/VSD usually has a sufficient intracardiac shunt, balancing systemic–pulmonary circulation and allowing delayed surgery. However, these infants are prone to heart failure and pulmonary hypertension, making surgical timing a clinical focus. Studies have shown that TGA/VSD infants should undergo surgical correction within 3 weeks of birth.^6^

This neonate had a large VSD and a thick PDA, presenting with heart failure (tachypnoea, feeding difficulty) early postnatally. Thus, our centre opted for earlier surgery. Preoperative evaluation showed normal left ventricular development/function and no coronary malformations. Despite small right ventricular volume (tricuspid Z-score −2.8), we assessed right ventricular function as follows: (i) The neonate was full-term, excluding prematurity-related right ventricular hypoplasia^7^; the RV and tricuspid valve were structurally normal, with good leaflet motion and insignificant tricuspid regurgitation, excluding right ventricular hypoplasia. (ii) Normal development of the aortic valve and aortic arch, satisfactory BP of four extremities, and no preoperative right ventricular hypertrophy suggested that the RV could tolerate adequate afterload and bear pulmonary circulation after anatomical correction. (iii) Preoperative left ventricular function was normal. (iv) Studies have demonstrated that those without RV-dependent coronary circulation, tricuspid annulus Z-score > −3, and the ratio of tricuspid-to-mitral valve diameter >0.5 are eligible for biventricular repair.^8^ (v) Palliative ASO plus pulmonary artery banding has been reported for TGA/VSD with right ventricular hypoplasia, with subsequent single-ventricle or biventricular correction based on right ventricular development.^9^ Based on the above reasons, we concluded that the patient’s RV was in a borderline state rather than right ventricular hypoplasia, and preoperative evaluation confirmed preserved right ventricular function. Given the high procedural complexity and mortality associated with staged repair, our centre decided to perform one-stage radical surgery for this patient. We planned to retain a 3–5 mm ASD to relieve right ventricular preload if the RV failed to tolerate the load intraoperatively.^9^ However, SpO_2_ was only 80%–90% (fraction of inspired oxygen: 100%) after weaning from CPB; retaining an ASD would exacerbate tissue hypoxia through right-to-left shunting, and the lack of volume load might delay right ventricular development. Thus, ASD closure was performed, and inhaled NO was used to reduce afterload and assist right ventricular recovery, with successful weaning from CPB.

The operation was uneventful, but global heart failure occurred immediately after surgery. Intraoperative assessment of coronary arteries and myocardial perfusion confirmed satisfactory coronary transplantation, excluding the most common coronary complications.^10^ Early low cardiac output was presumed related to CPB-related injury. After aggressive postoperative treatment, left ventricular function recovered rapidly on bedside echocardiography, whereas refractory right ventricular dysfunction persisted. Intraoperative right ventricular injury may result from multiple factors, including direct surgical trauma, reperfusion injury, ischaemia, or inflammation associated with CPB and intraoperative coronary injury with myocardial ischaemia.^10,11^ Studies have shown that increased right ventricular end-diastolic pressure can impair right coronary perfusion.^12^ In this case, the small preoperative RV and loss of interventricular shunting postoperatively led to elevated right ventricular end-diastolic pressure, impaired right coronary perfusion, and aggravated right ventricular dysfunction. Postoperative right ventricular dysfunction was ultimately attributed to the preoperative borderline RV. Conservative treatment led to gradual recovery, with TAPSE normalized at 6 months. This may be explained by the non-preservation of the ASD intraoperatively, which forced the RV to gradually adapt to postoperative pressure load transition and ultimately facilitated right ventricular catch-up growth. Despite a favourable outcome with successful biventricular repair, strict postoperative early treatment and circulatory management were required, with longer hospital stay and higher costs than the average for this disease.

Studies have shown that both left and right ventricular systolic and diastolic functions are significantly impaired after ASO. Left ventricular function can gradually recover during follow-up, whereas right ventricular dysfunction persists within 1 year after surgery.^11^ The slower recovery of the RV is related to preoperative ischaemia and hypoxia, more vulnerable myocardial protection of the anteriorly located RV, CPB-related ischaemia and inflammatory injury, direct surgical trauma, as well as postoperative pulmonary artery obstruction and coronary artery problems.^11^ Right ventricular systolic function is an independent predictor of prognosis after ASO^13,14^ and may affect the long-term exercise capacity of patients, indicating the need for long-term follow-up to confirm complete recovery of right ventricular function.

## Conclusions

A borderline RV is not an absolute contraindication to ASO. This strategy is reasonable and feasible in the presence of an intact tricuspid valve with normal leaflet excursion, full-term status excluding prematurity-related right ventricular hypoplasia, normal aortic arch configuration, absence of preoperative right ventricular hypertrophy, preserved left ventricular function, tricuspid annulus Z-score >−3, tricuspid-to-mitral annular diameter ratio >0.5, and no RV-dependent coronary circulation.

## Supplementary Material

ytag432_Supplementary_Data

## Data Availability

The data underlying this article are available in the article and in its online Supplementary material.

## Lead author biography

Rui Chen, MD, PhD, Chief Physician, Director of the Cardiac Center at Qingdao Women and Children’s Hospital. He specializes in surgical, interventional and hybrid procedures for congenital heart diseases and pediatric thoracic disorders, and has independently performed over 10 000 pediatric open-heart surgeries with cardiopulmonary bypass and congenital heart disease interventions.

## Notes

**Consent**: The patient provided informed consent in accordance with the COPE guidelines for this manuscript and associated images.
